# Size matters: Micro- versus nanobubbles in ultrasound imaging and therapy

**DOI:** 10.1126/sciadv.ads2177

**Published:** 2025-07-16

**Authors:** Mihir Sheth, Caed Knight, Qiang Wu, Alexandra Vasilyeva, Awaneesh Upadhyay, Luca Bau, Jia-Ling Ruan, Nicholas Ovenden, Eleanor Stride

**Affiliations:** ^1^ Institute of Biomedical Engineering, Department of Engineering Science, University of Oxford, Oxford, UK.; ^2^ Botnar Institute for Musculoskeletal Sciences, Nuffield Department of Orthopaedics, Rheumatology and Musculoskeletal Sciences, University of Oxford, Oxford, UK.; ^3^ Department of Oncology, University of Oxford, Oxford, UK.; ^4^ Department of Mathematics, University College London, London, UK.

## Abstract

This study investigates the reported ability of nanobubbles (<500 nanometers in diameter) to exhibit a comparable or superior acoustic response to microbubbles (>1 micrometer in diameter). Eight hypotheses were examined. Both the theoretical and experimental results supported only one hypothesis: The apparent echogenicity of nanobubbles under both linear and nonlinear imaging is due to the presence of preexisting microbubbles, which are not reliably detected by available nanoparticle sizing methods. There was no evidence to support the other hypotheses, although the possibility of microbubble formation due to bubble aggregation/coalescence or swelling due to gas absorption in vivo could not be completely ruled out. Nanobubbles may offer advantages in terms of circulatory stability and potential for therapeutic delivery compared with microbubbles, but these advantages must be weighed against the need to use higher bubble concentrations, higher ultrasound frequencies, and/or higher intensities to achieve equivalent imaging and/or therapeutic effects.

## Teaser

One microbubble for every few thousand nanobubbles will dominate ultrasound scattering at clinically relevant frequencies.

## INTRODUCTION

Microbubbles (1 to 10 μm in diameter) were initially developed as contrast agents for use in ultrasound imaging because of their echogenicity and have subsequently been investigated in a range of therapeutic applications (*1*, *2*). Reported drawbacks of microbubbles include their relatively short circulation half-lives and inability to extravasate, which puts them at a disadvantage compared with contrast agents used in magnetic resonance and computed tomography x-ray imaging. Consequently, various submicrometer particles have been investigated in both diagnostic and therapeutic applications, including superheated perfluorocarbon liquid droplets (*3*, *4*), solid gas-entrapping particles (*5*), and gas “nano” bubbles. Formulations of nanobubbles used in biomedical ultrasound applications are similar in composition to microbubbles but with diameters <500 nm. They have been widely explored in recent years and reported as being able to extravasate while remaining visible under both linear and harmonic ultrasound imaging, as well as offering greater stability and longer circulation times (*6*, *7*). Note that the term nanobubble is used here because of its now widespread use in the literature. It should be emphasized, however, that the formal definition of a nanoparticle stipulates a maximum dimension of <100 nm (*8*). Nanobubbles have also been reported as effective agents for numerous therapeutic applications, e.g., for blood-brain barrier (BBB) permeabilization, drug delivery, and mechanical ablation (*9*–*15*).

These reports are surprising, as theoretical calculations suggest that, for the same gas volume, submicrometer particles should not be able to exhibit comparable performance to microbubbles as either diagnostic or therapeutic agents under the same ultrasound exposure conditions. For example, the acoustic scattering cross section, *S*_scat_, for a single, spherical gas-filled bubble is defined as the ratio of the scattered acoustic power to the incident ultrasound intensity. For linear oscillations, i.e., small amplitudes, *S*_scat_ can be expressed as

$$S_{\text{scat}}=4\pi R_{0}^{2}\frac{\Omega^{4}}{{\left(\Omega^{2}-1\right)}^{2}+\delta^{2}\Omega^{2}}$$ (1)

where *R*_0_ is the initial bubble radius, Ω is the ratio of the driving frequency to the bubble’s undamped natural frequency, and δ is the damping ratio.

**Fig. 1.**
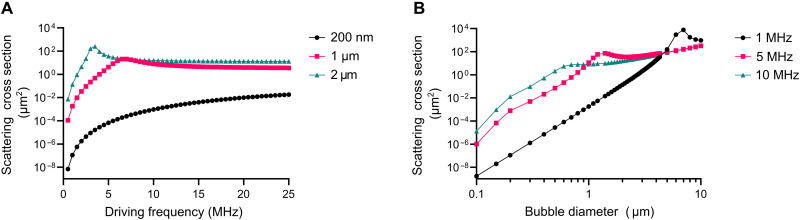
Theoretical acoustic scattering cross section. Variation in linear acoustic scattering cross section (Eq. 1) with (**A**) driving frequency for bubble diameters of 200 nm, 1 μm, and 2 μm for uncoated air bubbles in water and (**B**) bubble diameters for driving frequencies of 1, 5, and 10 MHz.

Even for an uncoated air bubble in water, which will oscillate with greater amplitude than a coated bubble, the linear scattering cross section for a nanobubble with a diameter of 200 nm is several orders of magnitude lower than that of a microbubble with a diameter of 1 μm (Fig. 1). Note that the term scattering cross section is used here for consistency with the literature. Strictly, Eq. 1 describes the ratio of the reradiated acoustic power to the incident intensity, which is usually substantially larger than the scattered power (*16*).

The nonlinear behavior of bubbles is also important in ultrasound imaging as it facilitates the use of techniques such as pulse inversion and/or amplitude modulation to maximize the contrast:background ratio (*17*). The amplitude of the nonlinear components of the radiated pressure from a bubble, however, will also be proportional to the bubble size, unless smaller bubbles exhibit substantially greater nonlinearity than larger ones. This possibility is explored below.

**Fig. 2.**
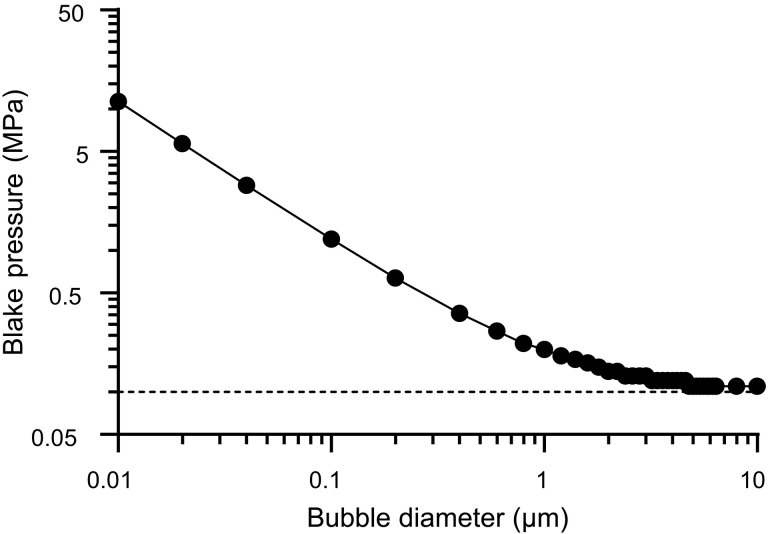
Theoretical Blake threshold. Variation in the Blake threshold (Eq. 2) with bubble diameter, indicating the minimum peak negative pressure required for cavitation of uncoated air bubbles in water (the dashed line indicates standard atmospheric pressure for reference).

When considering therapeutic applications, the Blake threshold provides a useful measure of the probability of cavitation. It is defined as the minimum pressure required to initiate unbounded growth of a bubble against surface tension (*18*)

$$\begin{array}{c} p_{B}=p_{o}\left\{\;1+\surd \left[\frac{32\sigma^{3}}{27p_{o}^{2}R_{0}^{2}\left(2\sigma +p_{o}R_{0}\right)}\right]\;\right\} \end{array}$$ (2)

where *p*_o_ is the ambient pressure and σ is the surface tension at the gas/liquid interface. As may be seen in Fig. 2, there is a very substantial difference in the pressure required to initiate cavitation from a microbubble compared with a nanobubble. Nevertheless, there are multiple reports of therapeutic effects being achieved at pressures <500 kPa, which are comparable or superior to those achieved with microbubbles. For example, Wu *et al.* (*9*) used 400-nm–diameter bubbles excited at 1 MHz and 1 W/cm^2^ intensity for gene delivery in vitro; Gattegno *et al.* (*19*) used 160-nm–diameter bubbles for BBB opening at 250 kHz, with peak negative pressures <200 kPa in a mouse model; and Suzuki *et al.* (*13*) used 500-nm bubbles at 1 MHz and 0.217 MPa “root mean squared average of sound peak pressure” for tumor ablation and immunostimulation, also in mice.

Several potential explanations for the seemingly paradoxical behavior of nanobubbles have been proposed in the literature (*20*, *21*). There is considerable variability in the definition of nanobubbles in published studies (diameters ranging from <100 nm to >1 μm). There is similarly a wide range of techniques used to determine their size and concentration (*22*, *23*) and a wide range of ultrasound parameters used across experiments, particularly frequencies from 0.25 to >20 MHz. The focus of this paper is the potential use of nanobubbles in human clinical applications and, thus, ultrasound frequencies <20 MHz (2 to 15 MHz for diagnostic applications and 0.5 to 3.5 MHz for therapeutic applications). Myers *et al.* (*22*) reported that published protocols for preparing nanobubbles generate mixtures of nano- and microbubbles and that, when the latter were removed from the suspensions, there was a negligible backscatter under B-mode ultrasound imaging (center frequency, 7 MHz; peak negative pressure, 330 kPa). This study aims to extend the work of Myers *et al.* and examine the following hypotheses:

1) The ultrasound scattering (linear and/or nonlinear) detected from suspensions thought to contain only nanobubbles (i.e., <0.5 μm in diameter) is in fact generated by a small proportion of preexisting microbubbles (i.e., >1 μm in diameter).

2) Coalescence and/or aggregation of nanobubbles upon exposure to serum results in the formation of microbubbles upon injection into tissue.

3) Coalescence and/or aggregation of nanobubbles upon exposure to ultrasound results in the formation of microbubbles during imaging.

4) Condensation of microbubbles during manufacture/purification by centrifugation results in superheated nanodroplets being present in the nanobubble suspensions.

5) There is nonlinear propagation through high-concentration bubble suspensions that generates harmonic content in the backscattered signal and so increases the contrast:background ratio of nanobubbles.

6) There are changes in the characteristic acoustic impedance of nanobubble suspensions at high bubble concentrations that produce strong ultrasound backscatter.

7) The nonlinear character of the bubble coating enhances the harmonic content in the backscattered signal and so increases the contrast:background ratio of nanobubbles.

8) The perfluorocarbon core of the bubbles absorbs gas from the surrounding liquid, causing an increase in bubble size.

## RESULTS

### Hypothesis 1: The ultrasound scattering detected from suspensions thought to contain only nanobubbles is in fact generated by a small proportion of preexisting microbubbles

The size distribution of the prepared bubble suspensions was measured before and after filtration and after dilution in phosphate-buffered saline (PBS). Before filtration, the mean diameter of the nanobubbles (P-NB) was 303 nm [95% confidence interval (CI): 295 to 312 nm; *n* = 9], and 121 (95% CI: 99 to 148; *n* = 9) bubbles per 10,000 were estimated to be larger than 1 μm (Table 1, table S1, and Fig. 3A). Filtration through a 0.45-μm polyvinylidene difluoride (PVDF) filter reduced the mean diameter by 87 nm (95% CI: 80 to 95 nm; *P* = 5.1 × 10^−9^, paired *t* test) to 216 nm (95% CI: 208 to 224 nm; *n* = 9). The fraction of microbubbles decreased 46-fold (95% CI: 19 to 112; *P* = 8.7 × 10^−6^, paired *t* test) to 2.6 (95% CI: 1.3 to 3.5; *n* = 9) per 10,000. These results are supportive of hypothesis 1 and consistent with the findings of Myers *et al.* The measurements also indicate that even mechanical filtration of the bubble samples did not completely eliminate microbubbles.

**Table 1.** Size distribution summary [geometric mean (95% CI) for concentrations and *P*(*D* >1 μm) and arithmetic mean (95% CI) for everything else] for each batch of the bubble suspensions that are unfiltered in PBS (P-NB), filtered through a 0.45-μm filter in PBS (P-NB-F). *P*, probability.

		Diameter							Concentration		
Filter	n*	*D*_10_ (nm)^†^	*D*_50_ (nm)^†^	*D*_90_ (nm)^†^	*D*[3,0] (nm)^‡^	Mean (nm)	SD (nm)^§^	CV (%)^¶^	Number (bubbles/ml)	Gas volume (μl/ml)	*P*(*D* >1 μm) (‱)^#^
–	9	151 (145–157)	253 (246–260)	515 (499–530)	447 (417–476)	303 (295–312)	194 (179–209)	64 (60–68)	9.18 × 10^11^ (7.11 × 10^11^–1.18 × 10^12^)	1.7 (1.4–2.2)	121 (99–148)
0.45 μm	9	135 (128–142)	204 (194–213)	312 (302–323)	291 (240–343)	216 (208–224)	87 (77–98)	40 (34–46)	1.27 × 10^11^ (7.68 × 10^10^–2.11 × 10^11^)	0.15 (0.099–0.24)	2.6 (1.3–5.5)

*Number of batches.

†Percentile diameters.

‡Volume-weighted mean diameter.

§Standard deviation.

¶Coefficient of variation.

#Fraction of bubbles larger than 1 μm in parts per 10,000.

**Fig. 3.**
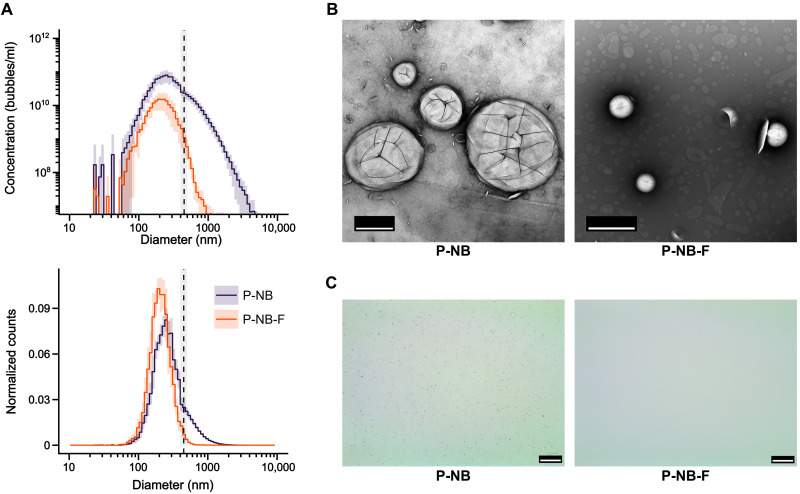
Bubble size distributions. (**A**) Composite size distributions of bubble suspensions before (P-NB) and after filtration through a 450-nm filter (dashed line) in PBS (P-NB-F), combining the measurements from ILM and EZS. Each line represents the average measurement from *n* = 9 separate batches, with the shaded area representing 1 SD. (**B**) Representative images from TEM and (**C**) bright-field optical microscopy. Scale bars, 500 nm (B) and 25 μm (C).

### Hypothesis 2: Coalescence and/or aggregation of nanobubbles upon exposure to serum results in the formation of microbubbles upon injection into tissue

**Fig. 4.**
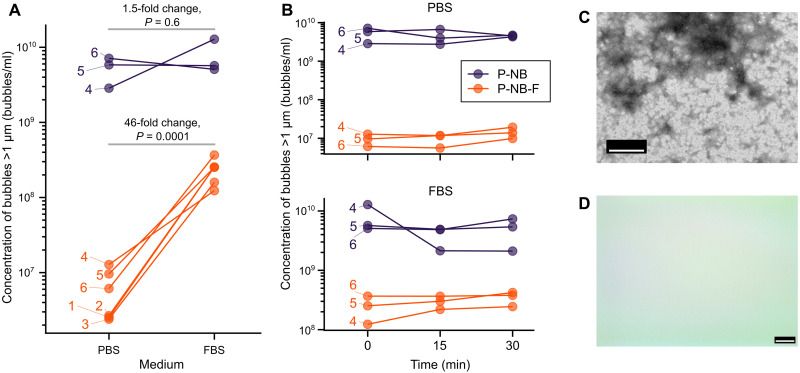
Effect of changing suspending medium. (**A**) Concentration of microbubbles (i.e., particles with diameter >1 μm) detected by EZS when P-NB and P-NB-F were suspended in PBS or 50% FBS at 37°C for samples from *n* = 6 separate batches shown by separate lines. (**B**) Change in microbubble concentration over 30 min in PBS and FBS for *n* = 3 batches. Representative images from (**C**) TEM and (**D**) bright-field optical microscopy of bubbles in FBS. Scale bars, 500 nm (C) and 25 μm (D). Further details can be found in the Supplementary Materials (table S1).

**Fig. 5.**
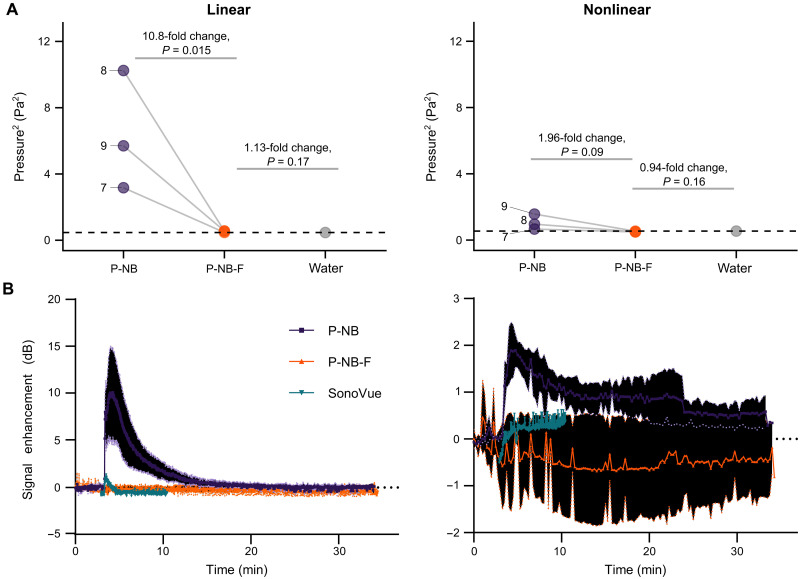
Ultrasound imaging of bubble suspensions in vitro and in vivo. (**A**) Square of the ultrasound pressure detected at the driving frequency (6 MHz corresponding to linear scattering) and second harmonic (12 MHz corresponding to nonlinear scattering) from bubble suspensions, before filtration in PBS (P-NB) and after filtration through a 450-nm filter in PBS (P-NB-F) against a PBS control in a tissue-mimicking phantom. For each suspension, *n* = 3 samples from separate batches (indicated by separate lines) were exposed to ultrasound for at least 50 pulses. P-NB suspensions were diluted so that the summed bubble volume was matched for P-NB and P-NB-F. For reference, the dashed line represents the scattering measured from PBS alone. (**B**) Linear B-mode and nonlinear contrast mode signal enhancement measured over time from a murine tumor following a tail vein injection of filtered (P-NB-F), unfiltered (P-NB), or SonoVue bubble suspensions and imaged at 18 MHz compared to preinjection images. Each line for P-NB and P-NB-F represents the mean average signal enhancement from three different mice, each receiving 100 μl of bubble suspension (each from a different batch). The dotted curves represent 1 SD.

The size distribution and concentration of bubbles larger than 1 μm before and after filtration (P-NB and P-NB-F respectively) of bubbles suspended in PBS or 50% fetal bovine serum (FBS) at 37°C were measured over time (Fig. 4, A and B, and fig. S3). Over the course of 30 min, the overall concentration remained substantially unchanged in all samples. In the filtered samples suspended in 50% FBS, however, the concentration of bubbles larger than 1 μm increased 46-fold (95% CI: 18 to 119; *P* = 0.0001, paired *t* test) to 2.2 × 10^8^ bubbles/ml (95% CI: 1.5 × 10^8^ to 3.3 × 10^8^ bubbles/ml; *n* = 6) compared to 4.8 × 10^6^ bubbles/ml (95% CI: 2.2 × 10^6^ to 1.0 × 10^7^ bubbles/ml; *n* = 6) in the filtered PBS samples. These changes cannot be explained by preexisting microbubbles in the serum (6.6 × 10^5^ bubbles/ml; 95% CI: 3.0 × 10^5^ to 1.4 × 10^6^ bubbles/ml; *n* = 5) and appear to have occurred very rapidly, before the first measurement time point. Similar effects were seen in both the filtered and unfiltered populations. Examination of bubble samples under electron microscopy, however, provided no evidence for coalescence or aggregation (Figs. 3, B and C, and 4, C and D), and there was no detectable signal from filtered bubbles in vivo (Fig. 5). Thus, hypothesis 2 is not directly supported by the results obtained but cannot be entirely ruled out.

### Hypothesis 3: Coalescence and/or aggregation of nanobubbles upon exposure to ultrasound results in the formation of microbubbles during imaging

Figure 5 and fig. S4 show the measured change in signal amplitude for linear and nonlinear ultrasound scattering from gas volume concentration–matched P-NB and P-NB-F flowing through a tissue-mimicking phantom. The unfiltered samples produced a detectable linear response, which was reduced 11-fold (95% CI: 3 to 38; *P* = 0.015, paired *t* test) in the filtered samples. The filtered samples produced negligible scattering at either the fundamental or higher frequencies. Similar findings were obtained from grayscale image intensity measurements taken with a commercial ultrasound scanner (fig. S5). These results suggest that the signal produced by the unfiltered samples was due to the presence of preexisting microbubbles in both linear and nonlinear imaging modes. If bubble coalescence or aggregation due to secondary radiation forces had been induced by ultrasound exposure, this should have produced a noticeable change in grayscale intensity over the course of the experiment. No such change was observed, either in the phantom or in vivo. These results suggest that hypothesis 3 is not valid and provide further support for hypothesis 1.

### Hypothesis 4: Condensation of microbubbles during manufacture results in superheated nanodroplets being present in the nanobubble suspensions

The results discussed in the preceding section for the filtered nanobubble samples (Fig. 5) suggest that vaporization is not occurring, as this should have resulted in visible microbubble activity. This is further supported by consideration of the pressure required for condensation of octafluoropropane (OFP) microbubbles to form nanodroplets. Mountford *et al.* (*24*) measured the pressure required to condense perfluorobutane microbubbles and reported values of 290 and 336 kPa, at 20°C, for 1,2-dipalmitoyl-sn-glycero-3-phosphocholine (DPPC)- and 1,2-dibehenoyl-*sn*-glycero-3-phosphocholine (DBPC)–based coatings, respectively. This can be considered an underestimate of the pressure required to condense perfluoropropane bubbles. The maximum hydrostatic pressure on the bubbles during centrifugation at 50*g* and 20°C is 108.5 kPa at a depth of 1.5 cm. Therefore, hypothesis 4 is not valid.

### Hypothesis 5: There is nonlinear propagation through high-concentration bubble suspensions that generates harmonic content in the backscattered signal and so increases the contrast:background ratio of nanobubbles

**Fig. 6.**
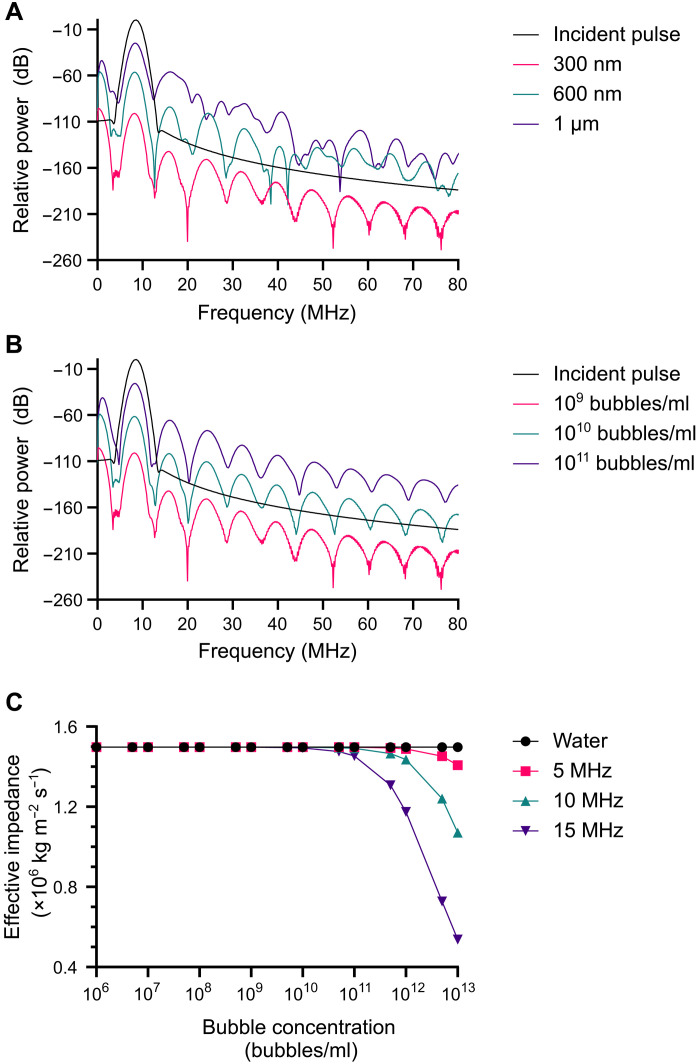
Numerical simulations of ultrasound propagation through bubble suspensions. Backscattered power relative to the incident pulse from simulations of the nonlinear propagation of an ultrasound pulse (six cycles, Gaussian enveloped, 8.5-MHz center frequency, and a peak negative pressure of 583 kPa), with a distance of 8 mm through suspensions of phospholipid-coated bubbles with (**A**) diameters from 0.3 to 1 μm and a fixed concentration of 10^9^ bubbles/ml and (**B**) a fixed diameter of 0.3 μm and different concentrations from 10^9^ to 10^11^ bubbles/ml. (**C**) Predicted change in the characteristic acoustic impedance of nanobubble suspensions (200-nm-diameter uncoated air bubbles) at different ultrasound frequencies with varying bubble concentrations.

Theoretical modeling predicted a substantial difference in the amplitude of the backscattered signal from micro- and nanobubble suspensions for both linear and nonlinear propagation (Fig. 6, A and B). The model in this case included a coating exhibiting nonlinear viscoelastic behavior. For nanobubbles, measurable scattering was only predicted at very high concentrations or frequencies above 15 MHz. These results were again in good agreement with the experimental ultrasound imaging measurements (Fig. 5), indicating that hypothesis 5 is not supported.

### Hypothesis 6: There are changes in the characteristic acoustic impedance of nanobubble suspensions at high bubble concentrations that produce strong ultrasound backscatter

Figure 6C shows the effect of increasing nanobubble concentration on the predicted characteristic acoustic impedance of a bubble suspension. Again, only at impractically high bubble concentrations (assuming a maximum starting concentration of 10^12^ bubbles/ml and a dilution factor of ~1000 following injection into the human bloodstream) is there a sufficiently large change in impedance to be potentially measurable. This indicates that hypothesis 6 is invalid.

### Hypothesis 7: The nonlinear character of the bubble coating enhances the harmonic content in the backscattered signal and so increases the contrast:background ratio of nanobubbles

**Fig. 7.**
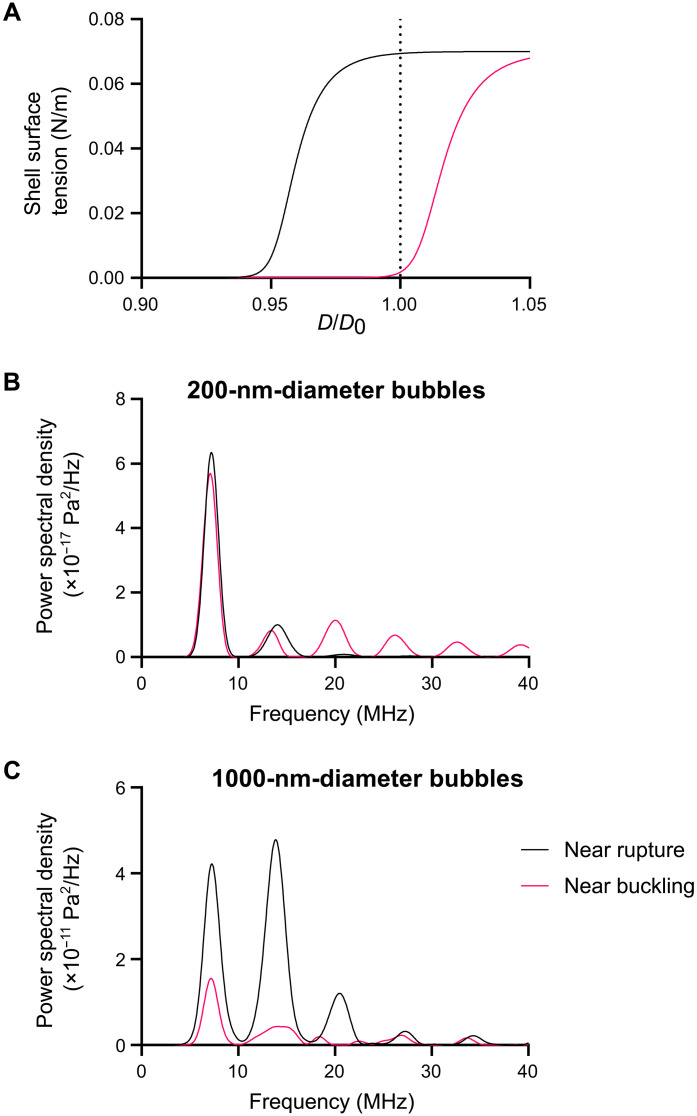
Frequency spectra of the radiated pressure spectra from micro- and nanobubbles showing the effect of coating nonlinearity. (**A**) Functional relationship between bubble diameter and surface tension for surfactant coatings that are initially either close to rupture or close to buckling. (**B** and **C**) Theoretical power spectral density of the radiated pressure from (B) nanobubbles (200 nm in diameter) and (C) microbubbles (1 μm in diameter) showing the effect of the coating state on the relative amplitudes of different spectral components.

Figure 7 shows the frequency content of the predicted radiated pressure from differently sized bubbles for different values of the coating parameters (Eq. 3) corresponding to different degrees of nonlinearity. As expected, the proportion of harmonic to fundamental content in the radiated pressure is affected by the selection of the coating parameters. The absolute magnitude of the signal from a 200-nm-diameter bubble, however, is substantially smaller (~6 orders of magnitude) than that from the 1-μm bubble. Thus, nanobubbles would not be expected to behave more nonlinearly than microbubbles or, hence, to be more visible using nonlinear imaging schemes. This is consistent with the predictions from Fig. 6 and the experimental measurements in vitro and in vivo (Fig. 5). Thus, the results suggest that hypothesis 7 is also invalid.

### Hypothesis 8: The perfluorocarbon core of the bubbles absorbs gas from the surrounding liquid, causing an increase in bubble size

**Fig. 8.**
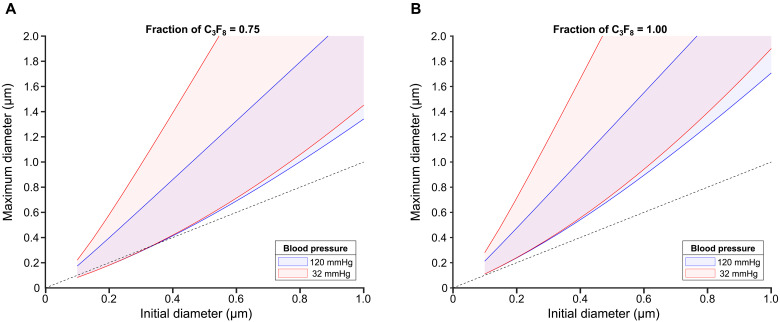
Predicted change in bubble size due to absorption of gases from the surrounding liquid. Maximum theoretical diameter of a C_3_F_8_ bubble over its expected lifetime caused by absorption of gases (oxygen and nitrogen) from the surrounding liquid for different initial bubble sizes, liquid pressures, and fractions of C_3_F_8_ in the bubble core, which was either (**A**) 0.75 or (**B**) 1.0. The shaded areas represent a range of interfacial tension at the bubble (1 to 25 mN/m).

Figure 8 shows the predicted maximum diameter of a C_3_F_8_ bubble over its expected lifetime caused by absorption of gases (oxygen and nitrogen) from the surrounding liquid for different initial bubble sizes depending on the fraction of C_3_F_8_ in the bubble core, the interfacial tension, and liquid pressure*.* The results indicate that it is feasible for bubbles to swell substantially at the pressure expected in capillaries (30 mmHg) if the coating reduces the interfacial tension to <5 mN/m and if the initial fraction of C_3_F_8_ is 100%. Swelling could therefore theoretically explain the reported measurements of scattering from nanobubble suspensions in vivo at frequencies <15 MHz. Neither the in vitro nor in vivo results obtained in this study, however, support this hypothesis as the filtered nanobubble samples did not generate a detectable ultrasound signal. Thus, there is no experimental evidence to support hypothesis 8, but it cannot be ruled out.

In addition to the experiments designed to test the stated hypotheses, nanobubble samples were exposed to ultrasound conditions more representative of therapeutic applications (0.5 MHz and pressures from 0.5 to 2 MPa). The bubble responses were recorded using ultrahigh-speed imaging. Filtration was again found to substantially reduce the observed bubble activity (movies S1 to S6), with the unfiltered samples being indistinguishable from a microbubble control sample (movie S7). A further control in PBS alone confirmed that there was no observable cavitation activity in the tube in the absence of bubble samples up to peak negative pressures of 2 MPa (movie S8). These findings are consistent with Fig. 2 and other studies of submicrometer cavitation nuclei (*3*, *5*), i.e., that smaller nuclei require greater peak negative pressures to generate observable bubbles. The presence of resolvable particles in movie S6, for P-NB-F, again suggests that filtration of the bubble samples does not completely eliminate microbubbles.

## DISCUSSION

### Limitations of this study

The dissolved gas content of the suspending liquid was not measured in the experiments. On the basis of the experimental protocol, it is expected that the suspending liquid would have been in equilibrium with the surrounding air (except where it was explicitly degassed), and care was taken to ensure that the experimental protocol was followed between repeats. Ideally, however, the gas content should be known. Similarly, the gas content of the bubbles themselves was not measured. The implications of these omissions are discussed further below.

The stability experiments were performed in either PBS or serum rather than whole blood. This was to reduce interexperimental variability that would be introduced by having to separate bubbles from blood cells for particle sizing. Previous work suggests that aggregation is most likely to be promoted by adsorption of proteins such as serum albumin (*25*), and similar effects have been reported for other lipid particles such as liposomes (*26*). The behavior of particles in biological media, however, can be very complex, and it is possible that interactions between nanobubbles and blood components not present in serum could promote additional aggregation/coalescence, although there was no evidence of this in the in vivo imaging results. Ultrasound-induced aggregation/coalescence was only investigated using relatively short pulses. Longer pulses might be expected to promote coalescence and/or rectified diffusion (*27*), although the majority of nanobubble imaging studies also use short pulses, e.g., (*28*, *29*), and in vitro experiments are performed in PBS in phantoms. Thus, aggregation/coalescence cannot explain the results reported in those papers.

The effect of size upon bubble stability was not explicitly investigated as there is already an extensive literature on the potential mechanisms by which nanobubbles (coated and uncoated) may exhibit superior stability to microbubbles (*30*, *31*), and this was outside the scope of this study. It is possible, however, that bubble size could affect coating structure and, hence, interfacial tension, gas permeability, and dynamic behavior. Previous work has shown that surface lipid packing differs with bubble size, composition, and manufacturing technique (*32*) and that it is a good predictor of bubble stability against gas diffusion. However, a subsequent study showed that there was no strong correlation between lipid packing and acoustic response (*33*). Because of their small size, it is not possible to use optical microscopy methods for lipid packing measurements on nanobubbles. Moreover, it would be necessary to confirm the gas content of specific bubbles to be certain that a valid comparison could be made between lipid packing and stability measurements.

An inherent challenge with nonlinear ultrasound imaging is the need to transmit and receive over different frequency ranges, while conventional ultrasound transducers have a limited bandwidth. It was therefore necessary to compromise between maximizing the transmitted amplitude and receiving sensitivity in the imaging experiments. This inevitably limits the degree to which nonlinear bubble dynamics can be excited and/or detected. Again, however, this challenge will also have been faced in previous studies using nonlinear modes for nanobubble imaging where nonlinear contrast enhancement was reported.

### Implications for contrast/cavitation agent selection

**Fig. 9.**
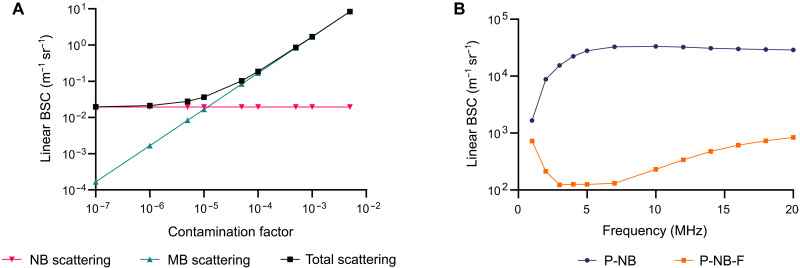
Effect of microbubble “contamination” on backscattering from nanobubble populations. (**A**) Theoretical linear BSC (Eq. 5) for a bubble population containing nanobubbles (NB; 200 nm in diameter) at a fixed concentration (10^9^ bubbles/ml) and microbubbles (MB; 1 μm in diameter) at different relative concentrations. The red and green lines show the contributions of the nano- and microbubbles, respectively, to the total backscatter of 7-MHz ultrasound, which is shown in black. The “contamination factor” is the ratio of the microbubble to nanobubble concentration. (**B**) Theoretical linear BSC for suspensions of filtered and unfiltered bubbles at different ultrasound frequencies based on the experimentally measured size distributions shown in Fig. 3.

Consistent with Myers *et al.*, both the theoretical and experimental results in this study suggest that the reported echogenicity of nanobubble suspensions is due to the presence of microbubbles. The results further suggest that these microbubbles are most likely present in the suspensions before application, because of the difficulties in completely segregating different bubble sizes from a polydisperse suspension. There is also the possibility that microbubbles may be formed by aggregation/coalescence of nanobubbles upon contact with biological fluids, as has been seen for other lipid structures (*26*), and/or absorption of gas from those fluids. As shown in Fig. 9A, only a very small fraction of microbubbles is needed to increase the effective linear backscattering coefficient (BSC) of an uncoated bubble population (Eq. 5) by several orders of magnitude. At 7 MHz, 1 microbubble per 10,000 nanobubbles would be sufficient to dominate the backscattered signal. This level of contamination is consistent with our measurements of the relative proportions of micro- and nanobubbles present in the experimental suspensions (121 and 2.6 microbubbles per 10,000, respectively, in the unfiltered and filtered suspensions; Table 1). These concentrations of microbubbles would be expected to be detectable by a standard imaging system, as a clinical dose of 10^8^ microbubbles diluted into 5 liters of blood yields an average in vivo concentration of 2 × 10^4^ microbubbles/ml, and this is sufficient to enhance vascular contrast. Figure 9B specifically shows the large difference in the expected linear BSC for filtered and unfiltered bubble suspensions based on the experimentally measured size distributions shown in Fig. 3. The contribution of the small number of microbubbles in the filtered suspensions at low frequencies is particularly noticeable. A similar effect would be expected for coated bubbles and the nonlinear content of the scattered signal (fig. S6).

Given the limitations of individual particle sizing devices (Materials and Methods), it is not unusual that this small number of microbubbles could go undetected when characterizing nanobubble suspensions. A similar problem is encountered in the presentation of microbubble characterization data. Typically, optical microscopy or electrical zone sensing (EZS) methods are used to size and count microbubbles, but neither of these methods will accurately register the presence of high concentrations of nanoparticles that are undoubtedly present in microbubble suspensions. The nanoparticles may comprise micelles of unincorporated lipids and, likely, gas-filled nanobubbles too. It is therefore important to carefully design characterization protocols to capture the full range of particle sizes potentially present in a suspension. Ideally, the protocol would also include a means of distinguishing gas-filled bubbles from other particles such as gas chromatography–mass spectroscopy, as recommended in (*21*). This would be particularly useful for assessing the impact of processing steps, such as mechanical filtration upon bubble destruction as well as particle concentration. In this study, it was only possible to match total particle volume based on the EZS/interferometric light microscopy (ILM) measurements rather than the total gas volume. There are, however, multiple studies in the literature reporting that filtered nanobubbles are still echogenic even at frequencies <10 MHz, e.g., (*34*).

Two key proposed advantages of nanobubbles over microbubbles are their stability in circulation and potential for extravasation (*35*). Multiple studies do suggest that the circulation times of particles in the 100- to 500-nm range may be longer than those in the micrometer range (*36*). Thus, there may well be a “sweet spot” in terms of bubble size to achieve sufficient echogenicity and adequate circulation times. There are, however, other factors affecting bubble stability, including their surface chemistry, which will affect adsorption of proteins, interaction with immune cells, and their rate of dissolution. There are similarly multiple factors affecting nanoparticle extravasation other than size (*37*–*39*). It is important when comparing microbubbles and nanobubbles that all these factors are controlled for, and this has not always been the case in published studies. In the past 10 years, there has also been substantial debate over the relevance of the enhanced permeability and retention effect in human tissue, which should be considered in selecting agents for different applications (*40*). It should further be noted that there are other agents such as liquid nanodroplets that can offer the same advantages in terms of circulatory stability and extravasation potential but do not require such high concentrations, frequencies, and/or pressures as nanobubbles to achieve comparable echogenicity and/or therapeutic effect to microbubbles (*41*–*43*).

One further potential advantage of nanobubbles is worthy of mention in relation to drug delivery applications. Similar to other nanoparticles, the higher surface area–to–volume ratio of a nanobubble to a microbubble suspension of equivalent gas volume may promote enhanced cellular interaction. This has been proposed as a potential mechanism to explain the mechanism of action of nanobubbles in, e.g., oxygen delivery (*44*). Considerable further investigation is required in this area, however, as the higher surface area–to–volume ratio of nanobubbles could, in fact, be a disadvantage if it increases the rate of gas diffusion. Moreover, it will still be very important to consider any disadvantages associated with the requirement for higher bubble concentrations, ultrasound frequencies, and/or intensities. There is also a pressing need for a consensus on nomenclature for micro- and nanobubbles, as the latter currently encompasses bubbles from 100 to 999 nm, yet bubbles in this range will exhibit substantially different acoustic responses (e.g., >4 orders of magnitude difference in BSC).

In summary, the results of this study support the findings of Myers *et al.* that the apparent echogenicity of nanobubble suspensions in both linear and nonlinear imaging is due to the presence of microbubbles. These microbubbles may be undetected by methods designed to characterize nanoparticles as it is very difficult to accurately size a highly polydisperse bubble suspension and also difficult to produce bubble suspensions containing only submicrometer bubbles even with mechanical filtration. The limitations of available bubble sizing methods need to be carefully considered in experimental design to avoid misinterpretation of results and to avoid errors in dose estimation. It is also possible that microbubbles could be formed by aggregation/coalescence of nanobubbles and/or swelling due to absorption of gases in vivo. However, no direct evidence of these phenomena occurring was obtained, and it is unlikely that these could explain published results in tissue phantoms. There was no evidence found to support the hypotheses of ultrasound-induced nanobubble coalescence/aggregation under the conditions tested or of nonlinear effects and/or high nanobubble concentrations being responsible for the reported acoustic responses in either diagnostic or therapeutic applications. While nanobubbles may offer advantages in terms of circulation time, extravasation, and/or cellular fusion, higher bubble concentrations, higher ultrasound pressure amplitudes, and/or higher frequencies will be required to elicit an acoustic response, and this may have important implications for collateral bioeffects.

## MATERIALS AND METHODS

### Experimental design

The study was designed to enable testing of the eight hypotheses set out in Introduction. This required a combination of theoretical modeling and practical experiments.

### Theoretical modeling

The oscillations of bubbles with diameters from 100 nm to 10 μm were modeled using a Rayleigh-Plesset–type equation (*45*)

$$\begin{array}{c} \rho_{L}\left(R\ddot{R}+\frac{3}{2}{\dot{R}}^{2}\right)+p_{o}+p_{A}\left(t\right)-p_{G0}{\left(\frac{R_{0}}{R}\right)}^{3\gamma }\\ +\frac{4\mu_{L}\dot{R}}{R}+\frac{4\eta_{s}\left(R\right)\dot{R}}{R^{2}}+\frac{2\sigma \left(R\right)}{R}=0\; \end{array}$$ (3)

where *R* is the instantaneous radius of the bubble and dots denote differentiation with respect to time, *R*_0_ is the initial bubble radius, ρ_L_ is the density of the surrounding liquid, μ_L_ is its viscosity, η_s_ is the viscosity of the bubble coating, σ is the tension of the gas/liquid interface, *p*_G0_ is the initial pressure of the gas inside the bubble, γ is the polytropic constant, and *p*_A_ is the time-varying pressure due to the ultrasound field. Both η_s_ and σ are modulated by the concentration of coating molecules on the bubble surface, which varies with *R*. The corresponding radiated pressure at a distance *r* from the bubble was approximated (*46*)

$$p_{\text{rad}}=\frac{\rho_{L}R\left(R\ddot{R}+2{\dot{R}}^{2}\right)}{r}\;$$ (4)

Both analytical and numerical solutions of Eqs. 3 and 4 were obtained, and these data were used to simulate the propagation of an ultrasound pulse through a bubble suspension using the methods described in (*47*). Because of the uncertainty associated with estimating coating parameters for submicrometer bubbles, uncoated gas bubbles of air in water were modeled in the majority of the simulations (i.e., ρ_L_ = 1000 kg m^−3^, μ_L_ = 0.001 Pa s, η_s_ = σ *=* 0, *p*_o_ = 0.1 MPa, *c*_L_ = 1500 m s^−1^, and the gas was modeled as ideal and undergoing adiabatic expansion and compression, γ = 1.4). Uncoated bubbles would be expected to undergo larger amplitude oscillations than coated bubbles and therefore represent an upper bound in terms of echogenicity. For the nonlinear propagation modeling, however, a bubble coating was included to capture any potential nonlinear effects arising from its presence [surface tension varied sigmoidally from 0 to 0.07 N m^−1^ with changing bubble size (*35*) as shown in Fig. 7A; η_s_ = constant = 4 × 10^−9^ kg s^−1^].

For reference, the linear BSC for a bubble population was also calculated as

$$\text{BSC}=\Sigma_{i=1}^{N}n\left(R_{i}\right)\frac{S_{\text{scat}\_i}}{4\pi }$$ (5)

where *n*(*R_i_*) describes the size distribution of the bubble population.

The pressure required to condense microbubbles into nanodroplets during centrifugation was calculated using the following equation

$$p\left(h\right)=p_{\text{ext}}+\int_{r_{0}}^{r_{0}+h}\rho_{L}\omega^{2}r\;dr=p_{\text{ext}}+\frac{1}{2}\rho_{L}\omega^{2}\left[{\left(r_{0}+h\right)}^{2}-{r_{0}}^{2}\right]$$ (6)

where ω is the centrifuge angular velocity calculated from the relative centrifugal force and rotor radius (56.6 rad/s), *r*_0_ is the distance of the meniscus from the center of the rotor (13.8 × 10^−2^ m), and *h* is the height of the liquid column (1.5 × 10^−2^ m) as measured from the experimental apparatus. The atmospheric pressure, *p*_ext_, was assumed to be 101.3 kPa, and the liquid density of the propylene glycol/glycerol/water mixture was used in the experiments (see below at 20°C), assuming no excess volume of mixing and the absence of free gas bubbles.

To investigate the possible swelling of nanobubbles due to absorption of gas from the surrounding liquid, we used equation 19 of the model presented by Kabalnov *et al.* (*48*, *49*) to calculate the maximum diameter that a bubble of a given initial size could attain under different conditions: different initial volume fractions of perfluoropropane (100 or 75%), different interfacial tensions at the bubble surface from 0 to 25 mN/m, and different liquid pressures and, hence, dissolved gas concentrations corresponding to the physiological range for differently sized blood vessels.

### Experimental investigations

#### Materials

1,2-dibehenoyl-sn-glycero-3-phosphocholine (DBPC), 1,2-dipalmitoyl-*sn*-glycero-3-phosphate (DPPA), 1,2-dipalmitoyl-*sn*-glycero-3-phosphoethanolamine (DPPE), and 1,2-distearoyl-*sn*-glycero-3-phosphoethanolamine-*N*-[methoxy(polyethylene glycol)-2000] (DSPE-mPEG 2000) were purchased from Avanti Polar Lipids Inc. (Alabaster, AL, USA). OFP was purchased from F2 Chemicals Ltd. (Preston, Lancashire, UK). PBS, FBS expected to contain albumin at a concentration of 0.025 g/ml (*50*), propylene glycol, and glycerol were purchased from Sigma-Aldrich Ltd. (Gillingham, Dorset, UK).

#### Nanobubble preparation

OFP nanobubbles (P-NB) were prepared as detailed in De Leon *et al.* (*23*). Briefly, DBPC, DPPA, DPPE, and DSPE-mPEG 2000 (6.1:1:2:1) were mixed with propylene glycol, glycerol, and PBS (1:1:8 by volume) in a glass vial (7 ml) and sonicated in a bath for 10 min at room temperature. The mixture (1 ml) was transferred to a 3-ml vial, and the headspace was evacuated and then filled with OFP and mechanically agitated for 39 s using a CapMix vial mixer (3M, St. Paul, MN, USA). The vial was inverted and centrifuged at 50 rcf (relative centrifugal force) for 5 min. The resulting suspension (100 μl) was withdrawn with a 21-gauge needle 5 mm from the bottom. For the sizing, stability, and in vitro experiments, the 100 μl was resuspended in 1.9 ml of PBS to form the P-NB suspension and mixed by inversion. The P-NB (1 ml) was pulled through sterile 450-nm PVDF filters (Thermo Fisher Scientific, UK) with the aim of removing microbubbles, i.e., bubbles with diameters >1 μm. These suspensions will be referred to hereafter as P-NB-F; 50% FBS was created by mixing FBS and PBS in a 1:1 ratio. P-NB-F-S consisted of 200 μl of the P-NB-F solution mixed in 1800 μl of 50% FBS. Three batches of P-NB, P-NB-F, and P-NB-F-S were made for these experiments from the same initial 100-μl suspension. For the in vivo experiments, P-NB refers to the 100 μl withdrawn from the vial after inverted centrifugation. For P-NB-F, 100 μl was withdrawn through a 450-nm PVDF filter from the vial after inverted centrifugation. The suspending media used for nanobubble preparation and in subsequent experiments were all stored in the same laboratory for multiple days and therefore expected to be in equilibrium with the ambient conditions in terms of their dissolved gas content and temperature. However, this was not explicitly verified.

#### Bubble size and concentration measurements

Previous studies have shown that all the available techniques for bubble characterization have their limitations (*51*–*53*). Multiple techniques were therefore used in this study.

ILM was conducted using a Videodrop system (Myriade, France). ILM also exploits Brownian motion to determine particle size, but, in this case, the motion of individual particles is tracked using the interference between transmitted and scattered light and then analyzed to obtain individual diffusion coefficients and, through the Stokes-Einstein equation, size. Single-particle techniques, in principle, have much higher resolution and can be more accurate than ensemble techniques such as dynamic light scattering. An additional advantage is that they directly measure number-weighted size distributions and concentrations. ILM can nominally size particles from ~10 nm to a few micrometers, although concentration measurements in the Videodrop are only accurate within a very narrow concentration range, requiring careful optimization of sample dilution, and detection sensitivity may drop substantially well before the limits of the nominal size range. For this study, 7-μl samples were injected into the measurement chamber, and particle tracking continued until at least 300 particles were tracked from each sample. P-NB, P-NB-F, and P-NB-F-S were diluted by 1:1000, 1:200, and 1:200 respectively in PBS. The light-emitting diode (LED) intensity and exposure time were adjusted to optimize the brightness of light reaching the detector.

EZS was conducted using a Multisizer 4e (Beckman Coulter, USA). EZS uses the Coulter principle, whereby a particle passing through a cylindrical aperture, through which an electrical current is also flowing, produces a temporary change in electrical impedance proportional to its volume. Like ILM, EZS is also a single-particle technique and, therefore, in principle, suitable for highly polydisperse samples. The main limitation is that the size range is determined by the choice of aperture diameter: The wider apertures required to measure large particles have a higher limit of detection as the signal-to-noise ratio is a function of both particle and aperture volume. For this study, we used a 20-μm aperture, which cannot detect particles smaller than 0.4 μm. For each measurement, a blank consisting only of 10 ml of filtered ISOTON II electrolyte solution was first measured. Five microliters of P-NB and 30 μl of P-NB-F and P-NB-F-S were then added to the ISOTON, and a new reading was taken, correcting for any particles found in the blank. On the basis of the ILM and EZS measurements, composite size distributions were created using EZS particle counts above 400 nm and rescaled ILM particle counts below 400 nm. The scale factor for the ILM particle counts was equal to the ratio of EZS to ILM counts between 400 and 500 nm.

#### Microscopy

Transmission electron microscopy (TEM) was conducted using a Tecnai T12 system (FEI, USA). Unlike the other methods described above, TEM enables direct visualization of bubbles at magnifications that allow a very wide range of sizes to be observed, but the number of bubbles that can be counted and sized is limited by the number of images that can reasonably be acquired and processed. It is also unknown to what degree sample processing alters bubble size and concentration. Gas bubbles present a challenge for electron microscopy because of the need to expose samples to a vacuum in most methods. For this study, the process developed by Owen and Stride (*54*) was used, whereby bubbles were stained with uranyl acetate to stabilize them before imaging. Samples were visualized at 80 kV, and images were acquired at ~0.8-μm underfocus with 15 e^−^/Å^2^ on an FEI Eagle charge-coupled device camera. For reference, images of the different bubble suspensions were also acquired using bright-field optical microscopy. Similar to TEM, the number of bubbles that can be measured is limited by practical considerations. However, the minimum resolvable size is restricted by the diffraction limit of light (250 nm), while for a TEM, the diffraction limit is slightly more than 0.23 nm (*55*). For this study, 5 μl of bubble suspension was pipetted into a hematocytometer (Bright-Line, Hausser Scientific, Horsham, PA, USA) and imaged on a DM500 optical microscope (Leica, UK) using a 40× objective.

#### Stability measurements

Stability over 30 min was recorded for P-NB and P-NB-F bubbles in PBS and FBS at 37°C. The solutions were placed in a water bath at 37°C, and sizing measurements were taken on the Multisizer every 15 min. The same batches were also placed on an optical stage (HWPT-96OL, Cell MicroControls, USA) attached to a microcontroller (mTC3 micro-Temperature Controller, Cell MicroControls, USA) to maintain the temperature at 37°C while images were taken on the optical microscope every minute.

#### Ultrasound imaging

Bubble suspensions were also imaged in a tissue-mimicking flow phantom with an embedded 1-mm-diameter cylindrical channel. The flow phantom was fabricated using a degassed biocompatible hydrogel, consisting of 1.5% w/v low–melting point ultrapure agarose gel (Invitrogen, Carlsbad, CA, USA). Two milliliters of diluted P-NB and P-NB-F suspensions was drawn through the channel in the flow phantom using a low-pulsatility peristaltic pump (MINIPULS Evolution, Gilson, Middleton, WI, USA) at a constant rate of 0.2 ml/min. The suspensions were diluted such that the gas-volume concentrations of each sample of P-NB were matched with its respective P-NB-F sample (~0.11 to 0.2 μl/ml). A diagnostic ultrasound imaging probe (L11-4 linear array, ATL Ultrasound, Bothell, WA, USA) connected to a Verasonics Vantage ultrasound research system (Verasonics Inc., Redmond, WA, USA) was positioned ~20 mm away from the channel in the phantom to record acoustic emissions. The array was calibrated as a function of frequency to correct for element-by-element sensitivity and diffraction effects so that bubble emissions could be reported in device-independent physical units (*56*). The recorded waveforms from each element were transformed into the frequency domain, corrected for the complex-valued element sensitivities, and retained for spectral analysis. Spectrograms of these acoustic emission data in the channel were calculated using a 10-μs window. The spectral features of the acoustic data were quantified by estimating the linear (drive frequency; *f*_0_ = 6 MHz) and nonlinear (harmonic; 2*f*_0_ = 12 MHz) component of the calibrated spectra. In addition, a different diagnostic ultrasound imaging system (L12-5 linear array, operated at 7 MHz using an iU22 imaging system, Philips, Bothell, WA, USA) was used to record both B-mode and contrast images from the channel for comparison between P-NB and P-NB-F. B-mode images were acquired at 16 frames/s and contrast mode images were obtained at 12 frames/s. The mechanical index was 0.1, and the distance from the probe face to the center of the channel was 3 cm. Data were collected for at least 80 frames per sample. A custom MATLAB (MathWorks, Natick, MA, USA) script was used to analyze the change in grayscale value from a region of interest (ROI) inside the channel before and after injection of the bubble suspensions. The results are included in the Supplementary Materials.

#### High-speed optical imaging

Ultrahigh-speed video footage of bubble samples under ultrasound exposure was captured using the setup described in (*57*). Briefly, bubble samples were pulled into a polyethylene tube of 180-μm inner diameter and 10-μm wall thickness (Advanced Polymers, Salem, NH, USA) submerged in a tank of degassed and deionized water. An objective lens with a numerical aperture of 0.8 and a working distance of 3 mm (LUMPLFLN 40XW, Olympus) was focused on the midplane of the tube and coupled to a high-speed camera (HPV-X2, Shimadzu, Tokyo, Japan). A single-element spherically focused ultrasound (FUS) transducer (0.5-MHz center frequency; H107, Sonic Concepts, Bothell, WA, USA) was also focused on the tube. The transducer was driven by a programmable arbitrary waveform generator (33220A, Agilent, Santa Clara, CA, USA), and the signal was amplified with a 300-W radiofrequency power amplifier (A300, ENI, USA) and sent to the FUS transducer via a 50-ohm matching network. The aperture and the geometric focus of the transducer were 64 and 63.2 mm, respectively. P-NB and P-NB-F samples and a control sample of PBS were exposed to ultrasound at 0.5 MHz and peak negative pressures up to 2 MPa. The high-speed camera was triggered using the output from the waveform generator. After a delay of 40 μs to allow for propagation of the ultrasound pulse to the focal region, the camera recorded 256 frames at up to 1 million frames per second. The ultrasound pulse length was adjusted so that bubbles were exposed to ultrasound throughout the imaging period. Digital images of 400 by 250 pixels were recorded; the image resolution was 0.7 μm per pixel, determined using the hemocytometer described above. Illumination was provided by a high-intensity light source (445 nm; SOLIS-1C, Solis High-Power LED, Thorlabs Ltd., Ely, UK).

#### Statistical analysis

After combining the data from ILM and EZS, statistical analysis was conducted on each batch. The 10th percentile, 50th percentile (median), and 90th percentile diameters, volume mean diameter (*D*_[3,0]_), mean diameter, standard deviation, coefficient of variation (CV), number concentration, gas concentration, and fraction of bubble >1 μm were calculated from the weighted size distribution after rescaling. The gas concentration was calculated from number concentration and *D*_[3,0]_. The CIs for the summary values in table S1 were calculated using critical values from *t* distributions with the appropriate degrees of freedom. All comparisons were conducted with two-sided paired *t* tests, and differences were deemed significant when *P* < 0.05.

#### In vivo experiments

All animal experiments were conducted by licensed personnel (PIL holders) in accordance with UK Home Office regulations and institutional guidelines, under the University of Oxford project license PPL PP8415318. Six female C57BL/6 mice (18 weeks old; average weight, 22.3 ± 2.2 g) were subcutaneously injected with 5 × 10^6^ UPPL1541 cells into the right flank. Eleven days after tumor inoculation (tumor size, 87.0 ± 19.1 mm^3^), contrast-enhanced ultrasound imaging was performed using a Fujifilm Vevo 3100 system equipped with an MH250 probe (18-MHz transmission frequency and 4% transmit power). After ~3 min of baseline data collection, ~100 μl of either P-NB or P-NB-F was administered as a bolus injection via a tail vein cannula. Images were captured at 1 frame per second for 30 min, with 30-dB contrast gain and 28-dB two-dimensional gain. For comparison, images were also obtained from a further animal using a commercial contrast agent SonoVue (Bracco Diagnostics, Geneva, Switzerland). ROIs were drawn outlining the tumor, and the mean linear and nonlinear power in the ROI was calculated as a function of time using the Vevo LAB software (Fujifilm, Japan) and plotted using GraphPad Prism. Signal enhancement was calculated relative to the average of the first 2 min of baseline data. Note that due to the limited injection volume allowed and the expectation that the tumor vasculature would be sparse compared to healthy tissue, bubble suspensions were not diluted for this experiment.
