# Supplementary material for: Size matters: Micro- versus nanobubbles in ultrasound imaging and therapy
Source: Sci Adv. 2025 Jul 16;11(29):eads2177. doi: 10.1126/sciadv.ads2177 (PMC12266114; doi:10.1126/sciadv.ads2177)
Supplement: Supplementary file 1 — Figs. S1 to S6 Table S1 Legends for movies S1 to S8 [file sciadv.ads2177_sm.pdf]

Science Advances
AAAS

Supplementary Materials for

Size matters: Micro- versus nanobubbles in ultrasound imaging and therapy

Mihir Sheth *et al.*

Corresponding author: Eleanor Stride, eleanor.stride@eng.ox.ac.uk

Sci. Adv. **11**, eads2177 (2025)
DOI: 10.1126/sciadv.ads2177

The PDF file includes:

Figs. S1 to S6
Table S1
Legends for movies S1 to S8

Other Supplementary Material for this manuscript includes the following:

Movies S1 to S8

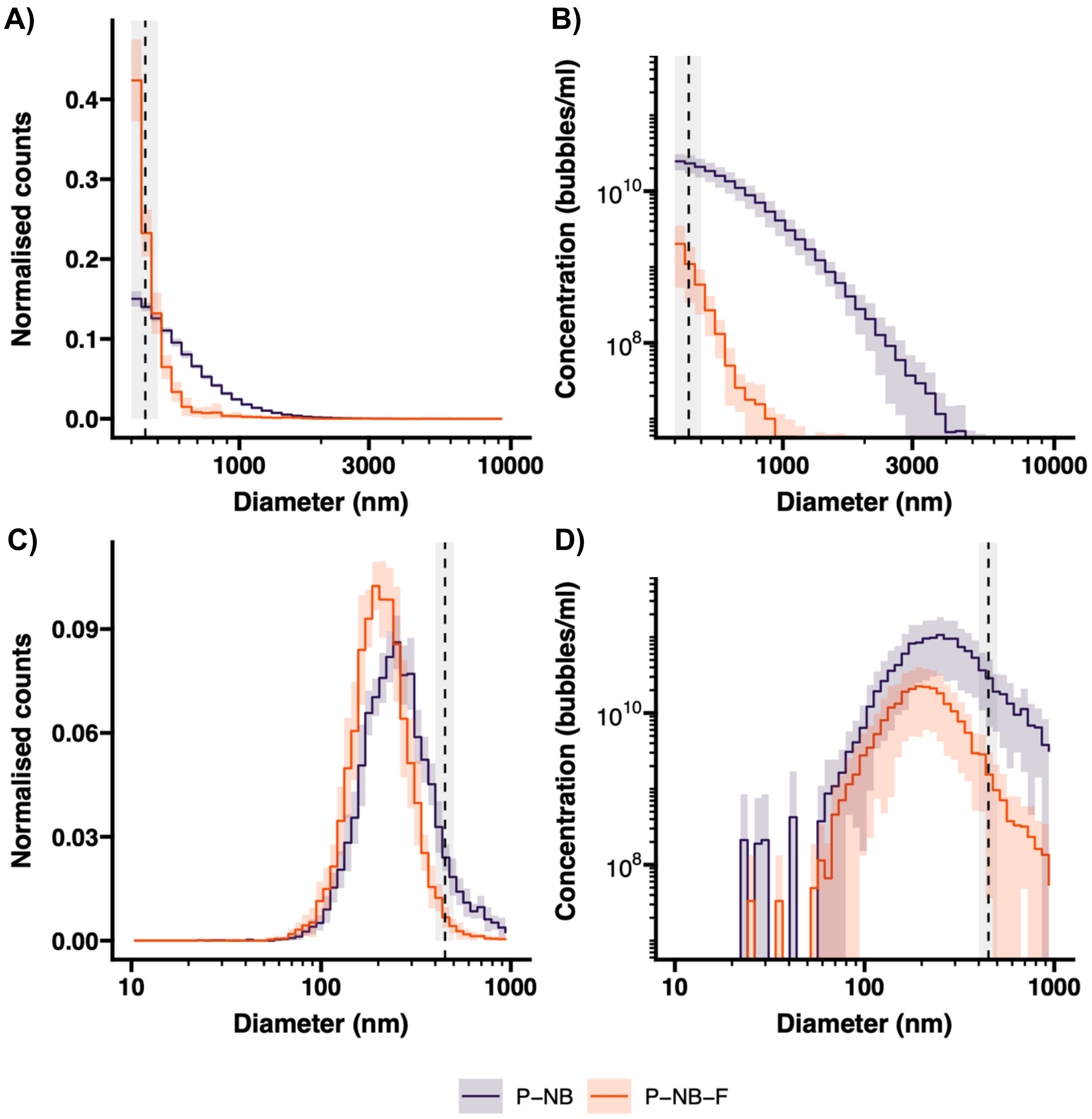

Fig. S1. Size distribution of P-NB and corresponding P-NB-F batches as measured by EZS in terms of normalised counts (A), and the total concentration of bubbles; and (B) as measured by ILM in terms of normalised counts (C) and the total concentration of bubbles (D). The solid lines show the mean values, while the shaded area represents one standard deviation (n = 9 separate batches). The dashed vertical line shows the 450 nm mark (the nominal mesh size of the filter).

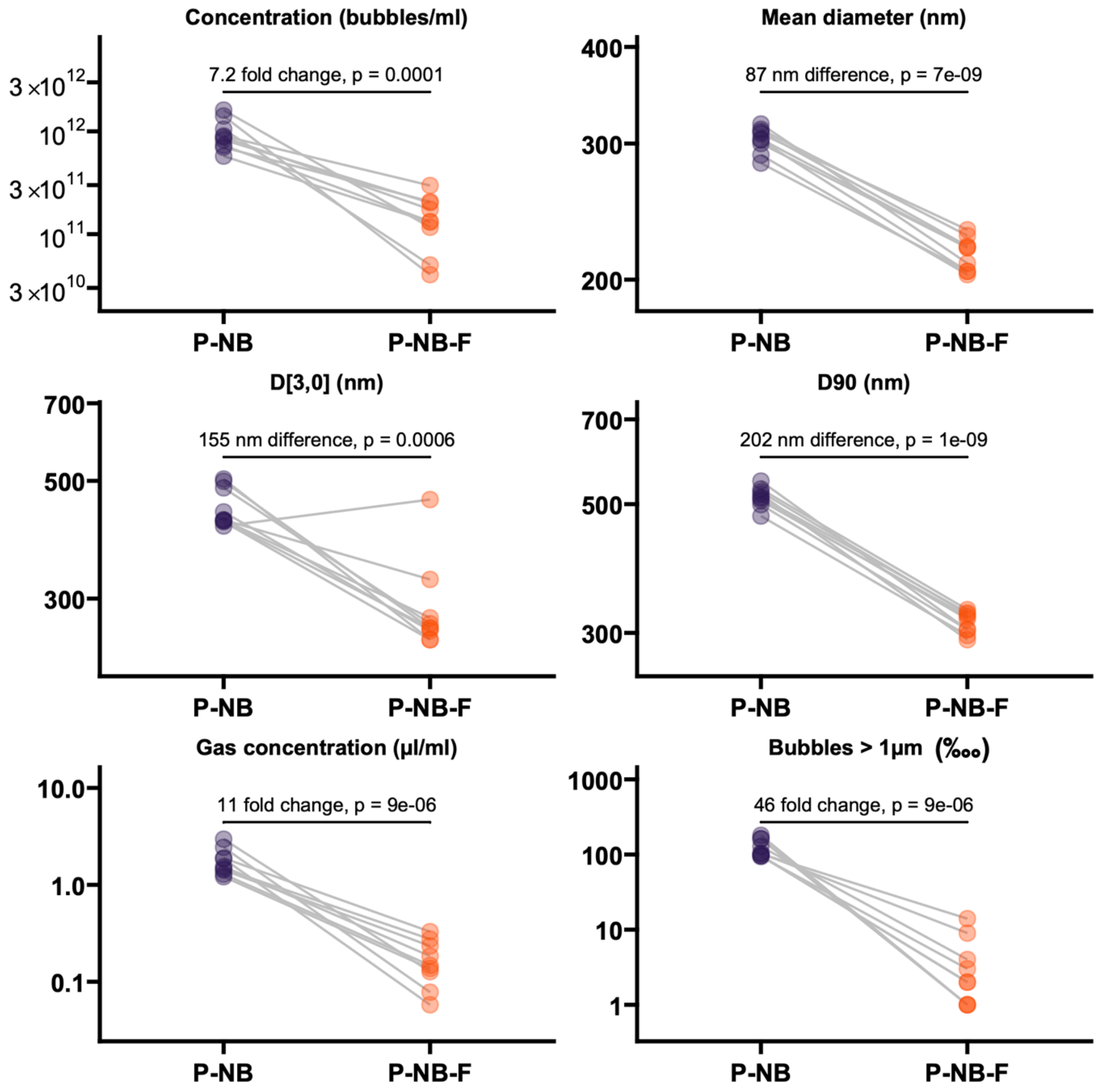

Fig. S2. Visual representation of the data shown in Table S1, with the particle concentration, mean diameter, $D_{[3,0]}$, D90, estimated gas concentration and particles having diameters > 1 µm (particles per 10,000) shown. Each line denotes a sample from the same batch before and after filtration.

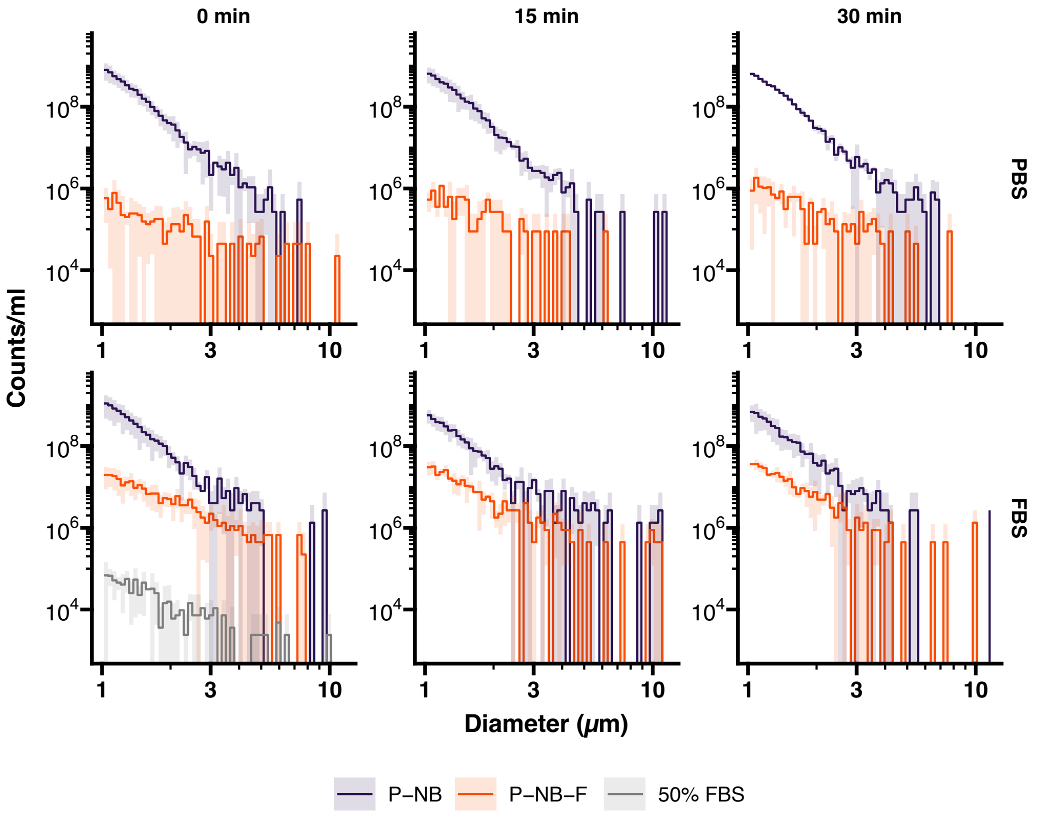

Fig. S3. Size Distribution of microbubbles (particles ≥ 1 μm) in PBS and 50% FBS at 37ºC as measured by EZS. The solid line shows the mean, while the shaded area represents one standard deviation from n = 3 samples from separate batches. The solid grey line shows the concentration of particles in 50% FBS.

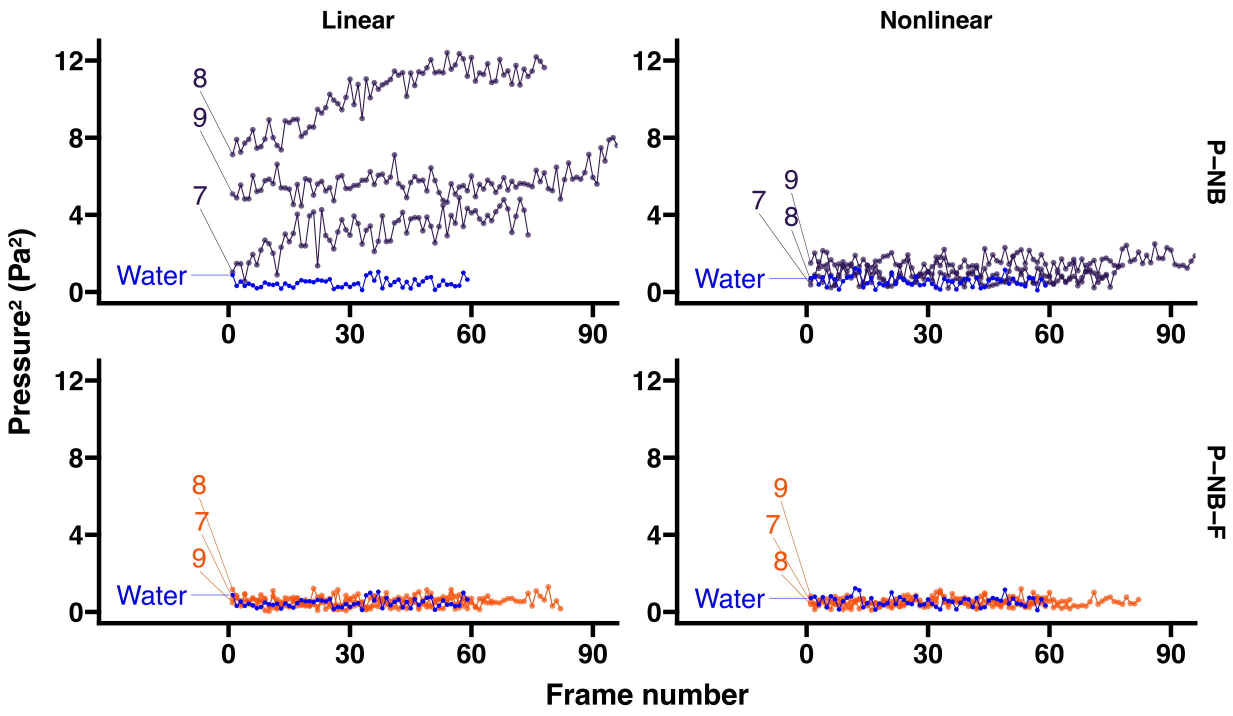

Fig. S4. Linear and non-linear ultrasound scattering measurements from P-NB and P-NB-F compared to a reference of water (PBS) in a tissue mimicking phantom. Lines indicate measurements from 3 samples from 3 separate batches.

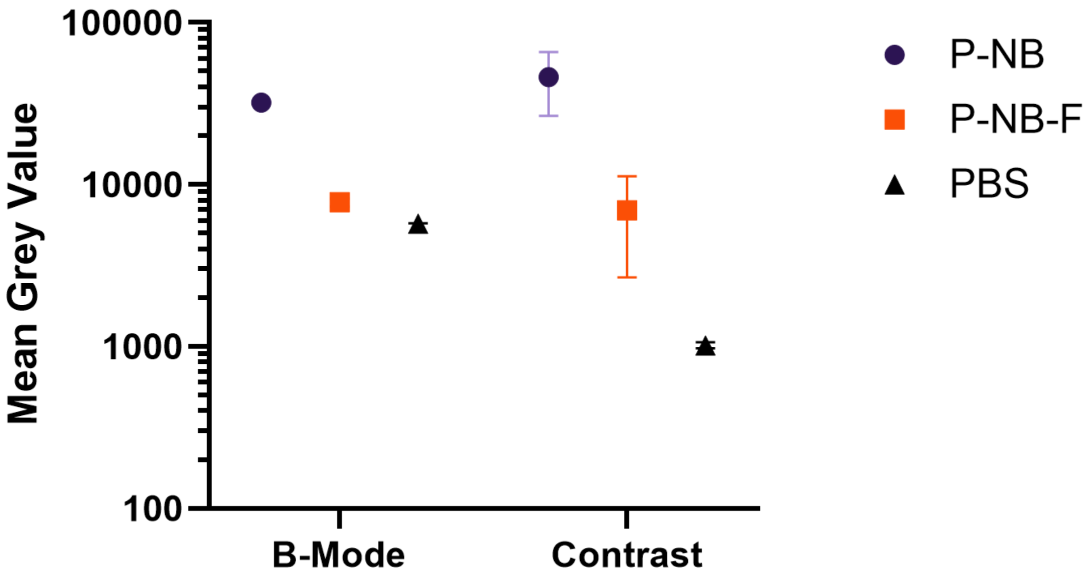

Fig. S5. Ultrasound imaging of bubble suspensions. Mean and Standard Deviation of the greyscale intensity values measured from ultrasound images of bubble suspensions, before filtration in PBS (P-NB), and the same batches after filtration through a 450 nm filter in PBS (P-NB-F), and from a control solution of PBS, in a tissue mimicking phantom using B-mode and contrast mode (pulse centre frequency 7 MHz, Mechanical Index 0.1). n=3 batches were tested for all samples, except for P-NB-F in B-Mode where n=2 batches were tested.

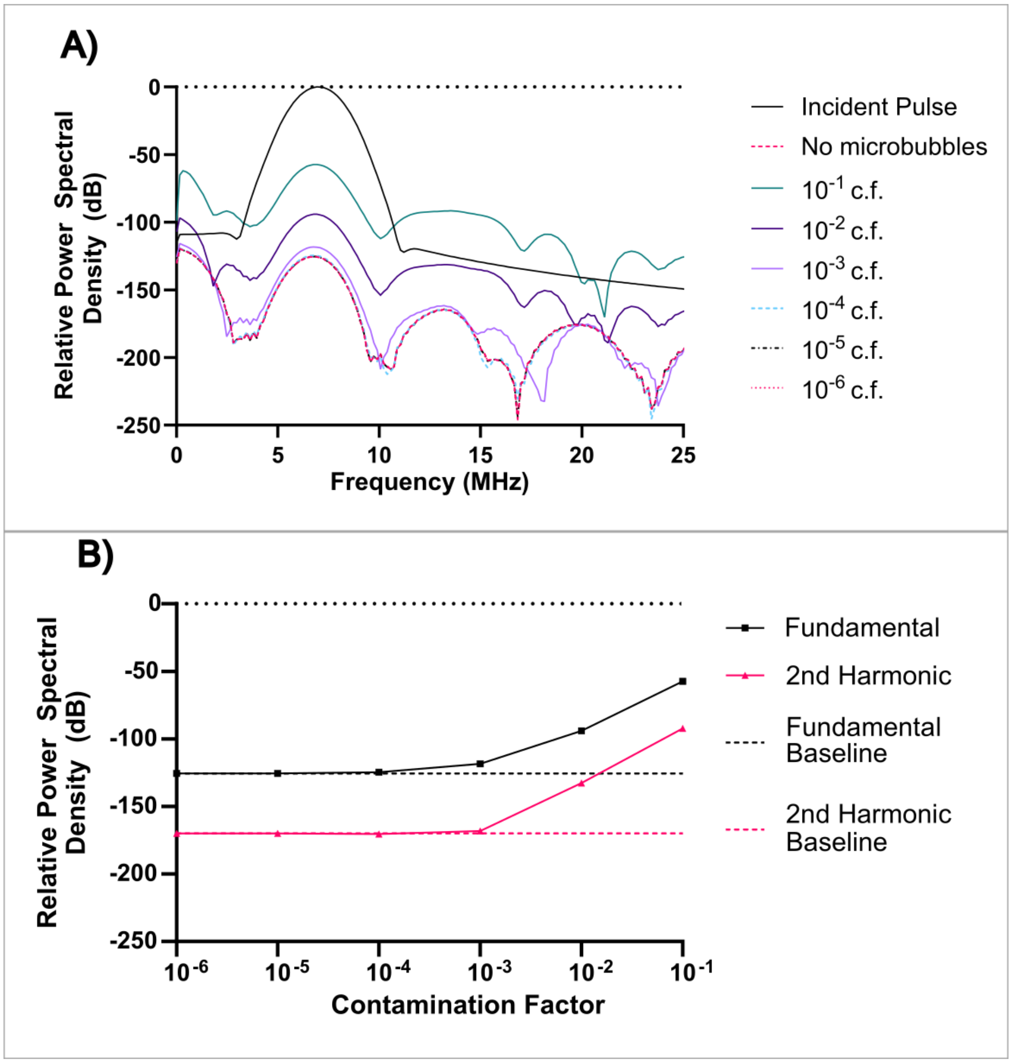

Fig. S6. Effect of microbubble "contamination" on non-linear scattering from nanobubble populations. (A) Power spectral density normalized to the incident pulse (7 MHz centre frequency) for different contamination factors (c.f. as defined in Fig. 9) from simulated non-linear scattering from bubble populations containing different relative concentrations of micro (1 μm diameter) and nanobubbles (200 nm diameter). (B) Relative power spectral density of the fundamental and second harmonic components from (A).

Table S1. Size Distribution Summary for each batch of the bubble suspensions that are unfiltered in PBS (P-NB), filtered through a 0.45 µm filter in PBS (P-NB-F), and filtered through a 0.45 µm filter in 50% FBS (P-NB-F-S).

		Diameter							Concentration		N		
**Batch**	**Filter**	**D_{10} (nm)[a]**	**D_{50} (nm)[a]**	**D_{90} (nm)[a]**	**D[3,0] (nm)[b]**	**Mean (nm)**	**SD (nm)[c]**	**CV (%)[d]**	**Number (bubbles/ml)**	**Gas volume (µl/ml)**	**Multisizer[e]**	**Videodrop[e]**	**P(D > 1 µm) [95% C.I.] (‱)[f]**
1	—	163	264	514	422	309	177	57	8.28×10^{11}	1.4634	74765	1001	97 [79-115]
1	0.45 µm	150	222	325	326	232	89	38	2.02×10^{11}	0.2755	3820	1015	2 [1-3]
2	—	158	261	507	419	304	177	58	8.77×10^{11}	1.5405	74131	1023	96 [78-115]
2	0.45 µm	140	210	320	251	220	78	36	1.74×10^{11}	0.1844	2344	1007	2 [1-4]
3	—	148	252	511	420	300	180	60	7.31×10^{11}	1.283	64116	1049	94 [77-112]
3	0.45 µm	136	206	317	264	220	85	38	1.33×10^{11}	0.147	2566	1005	3 [1-4]
4	—	140	236	477	411	283	180	63	1.41×10^{12}	2.4362	101833	1022	102 [79-124]
4	0.45 µm	126	189	297	461	205	120	59	4.02×10^{10}	0.078	556	1032	14 [7-23]
5	—	152	255	520	437	303	191	63	1.61×10^{12}	2.9519	138819	1024	128 [102-153]
5	0.45 µm	128	193	292	261	205	82	40	1.17×10^{11}	0.1275	1270	1030	4 [2-7]
6	—	138	241	499	422	290	186	64	1.05×10^{12}	1.8596	82770	1029	104 [82-125]
6	0.45 µm	121	189	304	276	203	95	46	5.01×10^{10}	0.0577	888	1057	9 [5-15]
7	—	153	255	548	504	318	225	71	5.74×10^{11}	1.2129	60417	1018	180 [150-208]
7	0.45 µm	134	197	304	251	210	75	36	1.31×10^{11}	0.1376	1156	1005	1 [0-2]
8	—	154	253	525	499	311	219	71	9.03×10^{11}	1.8887	85631	1040	161 [133-189]
8	0.45 µm	133	211	323	264	221	82	37	2.97×10^{11}	0.3287	4949	1034	1 [0-1]
9	—	150	259	531	485	313	214	68	7×10^{11}	1.4203	67241	1005	163 [134-190]
9	0.45 µm	144	217	329	269	228	78	34	2.07×10^{11}	0.2343	2783	1048	1 [0-2]

[a] Percentile diameters.

[b] Volume-weighted mean diameter.

[c] Standard deviation.

[d] Coefficient of variation.

[e] Number of particles counted.

[f] Fraction of bubbles larger than 1 µm in parts per 10,000.

List of supplementary movie files

Movie S1. High-speed microscopy footage of an unfiltered nanobubble suspension exposed to ultrasound. The centre frequency, pulse length and peak negative pressure of the driving pulse were 0.5 MHz, 50 cycles and 0.5 MPa respectively.

Movie S2. High-speed microscopy footage of an unfiltered nanobubble suspension exposed to ultrasound. The centre frequency, pulse length and peak negative pressure of the driving pulse were 0.5 MHz, 50 cycles and 1.0 MPa respectively.

Movie S3. High-speed microscopy footage of an unfiltered nanobubble suspension exposed to ultrasound. The centre frequency, pulse length and peak negative pressure of the driving pulse were 0.5 MHz, 50 cycles and 1.5 MPa respectively.

Movie S4. High-speed microscopy footage of a filtered nanobubble suspension exposed to ultrasound. The centre frequency, pulse length and peak negative pressure of the driving pulse were 0.5 MHz, 50 cycles and 0.5 MPa respectively.

Movie S5. High-speed microscopy footage of a filtered nanobubble suspension exposed to ultrasound. The centre frequency, pulse length and peak negative pressure of the driving pulse were 0.5 MHz, 50 cycles and 1.0 MPa respectively.

Movie S6. High-speed microscopy footage of a filtered nanobubble suspension exposed to ultrasound. The centre frequency, pulse length and peak negative pressure of the driving pulse were 0.5 MHz, 50 cycles and 1.5 MPa respectively.

Movie S7. High-speed microscopy footage of a filtered nanobubble suspension exposed to ultrasound. The centre frequency, pulse length and peak negative pressure of the driving pulse were 0.5 MHz, 50 cycles and 0.5 MPa respectively.

Movie S8. High-speed microscopy footage of phosphate buffered serum exposed to ultrasound. The centre frequency, pulse length and peak negative pressure of the driving pulse were 0.5 MHz, 50 cycles and 2.0 MPa respectively.
